# Microstrip Patch Sensor for Salinity Determination

**DOI:** 10.3390/s17122941

**Published:** 2017-12-18

**Authors:** Kibae Lee, Arshad Hassan, Chong Hyun Lee, Jinho Bae

**Affiliations:** 1 Research Institute, Kyungwon Co. Ltd., Siheung 15084, Korea; kibae0211@gmail.com; 2 Department of Ocean System Engineering, Jeju National University, Jeju 690-756, Korea; baejh@jejunu.ac.kr; 3 Department of Electrical Engineering, National University of Computer and Emerging Sciences, Foundation for Advancement of Science and Technology (NUCES-FAST), H 11/4, Islamabad 44000, Pakistan; arshad.hassan@nu.edu.pk

**Keywords:** microstrip patch sensor, salinity, relative permittivity, resonance frequency, sensitivity

## Abstract

In this paper, a compact microstrip feed inset patch sensor is proposed for measuring the salinities in seawater. The working principle of the proposed sensor depends on the fact that different salinities in liquid have different relative permittivities and cause different resonance frequencies. The proposed sensor can obtain better sensitivity to salinity changes than common sensors using conductivity change, since the relative permittivity change to salinity is 2.5 times more sensitive than the conductivity change. The patch and ground plane of the proposed sensor are fabricated by conductive copper spray coating on the masks made by 3D printer. The fabricated patch and the ground plane are bonded to a commercial silicon substrate and then attached to 5 mm-high chamber made by 3D printer so that it contains only 1 mL seawater. For easy fabrication and testing, the maximum resonance frequency was selected under 3 GHz and to cover salinities in real seawater, it was assumed that the salinity changes from 20 to 35 ppt. The sensor was designed by the finite element method-based ANSYS high-frequency structure simulator (HFSS), and it can detect the salinity with 0.01 ppt resolution. The designed sensor has a resonance frequency separation of 37.9 kHz and reflection coefficients under −20 dB at the resonant frequencies. The fabricated sensor showed better performance with average frequency separation of 48 kHz and maximum reflection coefficient of −35 dB. By comparing with the existing sensors, the proposed compact and low-cost sensor showed a better detection capability. Therefore, the proposed patch sensor can be utilized in radio frequency (RF) tunable sensors for salinity determination.

## 1. Introduction

Low-salinity water inflow into seawater has been a problem in the mass stranding of conch, abalone, farming fishes, etc. Rapid determination of salinity is required in order to cope with the inflow, which is usually carried out by using conductivity-temperature-depth (CTD) and satellite measurements [1,2,3,4]. The CTD has good accuracy, but the bulky CTD requires a great deal of human resources and time to measure the salinity. The CTD also has difficulty in the measurement of seawater surface properties. Satellites can measure only the salinity of seawater surface, and has poor spatial resolution, making accurate salinity determination in small areas impossible.

To overcome these difficulties, a small and portable salinity sensor has been developed [5,6]. The portable sensor uses conductivity measurement for sensing salinity with the aid of an internal temperature sensor. To monitor salinity levels in groundwater by using conductivity measurement, researchers have demonstrated a copper solenoid coils-based sensor [7]. To achieve sufficient accuracy, they have gone through several tests for calibration, which is a time-consuming process. Two new sensors based on the alteration of electromagnetic field for measuring water conductivity are reported, which avoids periodic calibration [8]. However, these sensors have lower sensitivity and accuracy as compared to CTD, and also suffer from erroneous data due to heat and small changes in the position of the sensor. Recently, research on detecting liquid type and concentration by using microwave frequency characteristics according to permittivity of the liquid has been reported [9,10]. Among microwave based designs, microstrip patch antennas are low-cost, low profile, lightweight, and easy to fabricate. Furthermore, they have narrow bandwidth and sharp frequency response as well, which is useful for measuring a physical quantity when it is used as a sensor. Because of these advantages, microstrip patch antennas have received a great deal of attention and microstrip patch designs have recently been reported for the measurement of salinity [11,12]. For detection of sugar and salt content in water, a Taconic TLY-5 substrate and a relatively large rectangular patch of 57.6 mm × 47.6 mm and 39.41 mm × 30.8 mm were used, respectively. To supply the signal to microstrip patch sensor, a direct contact with the patch by using a coaxial probe feeding technique was adopted [11]. For L-band remote sensing of soil and sea surface salinity, a microstrip design based on a stacked-patch array to feed a large mesh antenna and a large seven-element stacked patch array suitable for space applications were reported [12].

In this paper, a compact and accurate salinity determination sensor is proposed. In contrast to the existing sensors which use current to conductivity change, the proposed sensor uses the change in the resonance frequency as a function of the medium permittivity. In other words, the sensor is designed to have a particular resonance frequency according to unique permittivity, for which the relationship between the resonance frequencies with the permittivity can be approximated by a linear function. For actual sensor design, a microstrip patch design is adopted, which results in a small size and low-cost sensor. ANSYS high-frequency structure simulator (HFSS) is used to design and verify the proposed design. Finally, the sensor is fabricated with commercial parts, and the proposed design is verified via an experiment which is conducted with distilled water having different NaCl concentrations.

The rest of the paper is organized as follows. In Section 2, the proposed microstrip patch sensor design and analysis is presented. The fabrication process of the salinity sensor and the experimental setup for characterization is described in Section 3. The performance evaluation of the proposed sensor through simulation and experimental results are discussed in Section 4. Section 5 presents the conclusion of the paper.

## 2. Sensor Design and Analysis

**Figure 1:**
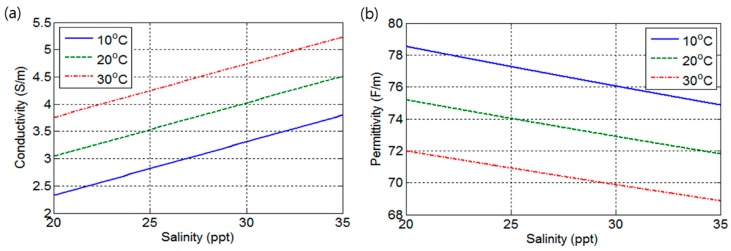
(**a**) Conductivity of seawater according to salinity; (**b**) Permittivity of seawater according to salinity.

It is well known that the conductivity and permittivity of seawater changes according to changes of salinity and temperature. The surface temperature of seawater ranges from 10 to 30 °C, but temperature below the permanent thermocline remains at approximately 10 to 15 °C. At fixed temperature, permittivity and conductivity change according to salinity with constant rate, so the salinity can be measured with the help of permittivity or conductivity value. However, the temperature change can lead to inaccuracy in the measurement of the salinity. To obtain accurate salinity determination, a Pt-resistance thermometer or thermistor is usually used for measuring temperature and compensating bias of the salinity according to the temperature. The first-order approximate expression for conductivity σ [13] can be expressed as follows:

(1) σ=0.098S+0.0716T−0.348

where *S* and *T* represent salinity and temperature, respectively. Similarly, the permittivity based on Wentz equation [14,15,16] can be expressed as follows:

(2) $$\varepsilon =\frac{3.70886\times 10^{4}-8.2168\times 10T}{4.21854\times 10^{2}+T}\cdot exp\left(b_{0}S+b_{1}S^{2}+b_{2}TS\right)$$

where $b_{0},\;b_{1},$ and $b_{2}$ are fitting coefficients determined as $b_{0}=-3.56417\times 10^{-3}$, $b_{1}=4.74868\times 10^{-6}$, and $b_{2}=1.15574\times 10^{-5}$. Using Equations (1) and (2), conductivity and permittivity changes according to temperature and salinity can be obtained as shown in Figure 1. It can be observed that the slopes of permittivity and conductivity at fixed temperatures of 10 °C, 20 °C, and 30 °C are 0.244 and 0.098, respectively. Permittivity and conductivity variations at fixed temperature are 3.67 and 1.47, respectively. These results indicate that permittivity change is 2.5 times more sensitive compared to conductivity change. Based on this fact, better salinity determination performance can be expected by the use of permittivity changes.

**Figure 2:**
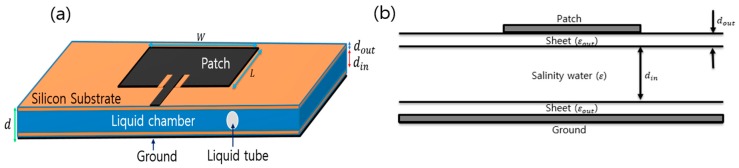
(**a**) Geometry of the microstrip patch resonator; (**b**) Structure of the proposed salinity sensor.

For salinity determination, a microstrip patch antenna is proposed, which is shown in Figure 2a. In the proposed design, both patch and ground plane are placed on the silicon substrates and a liquid chamber is made between the silicon substrates. Consequently, the proposed sensor can detect salinity with better accuracy by examining the resonance frequencies caused by different salinities.

The resonance frequency depends on width *W*, length *L*, relative permittivity $\varepsilon_{r}$, and thickness *d* of the microstrip patch antenna [17,18]. Once the dimension of the patch is determined, the resonance frequency only depends on $\varepsilon_{r}$. The proposed sensor is composed of a thin metal radiator patch and a ground plane attached to a dielectric substrate. The empty space between the two patches is filled with seawater. The cross-section of the proposed sensor is shown in Figure 2b, which shows three dielectric layers.

The relative permittivity of the sensor depends on the thickness and the relative permittivity of the individual layers. To obtain good radiation efficiency, the thickness *d* of the sensor is calculated as follows [17]:

(3) $$d=d_{in}+2d_{out},\;d\ll \lambda_{0}$$

where $\lambda_{0}$ is the free space wavelength at the resonance frequency. The thickness (*d*) should be much smaller than $\lambda_{0}$ for impedance matching between sensor and transmission line [17]. In this paper, $\lambda_{0}$ is 100 mm at the maximum resonance frequency of the designed sensor. Additionally, the designed sensor thickness (*d*) is 5.2 mm, which is very thin compared to $\lambda_{0}$. The relative permittivity ($\varepsilon_{r}$) of the proposed sensor can be determined by the following [17,18,19,20]:

(4) $$\varepsilon_{r}=\frac{d}{\frac{d_{in}}{\varepsilon }+2\frac{d_{out}}{\varepsilon_{out}}}$$

where $d_{in}$ and ε are the height of the liquid chamber and the relative permittivity of seawater, respectively, and $d_{out}$ and $\varepsilon_{out}$ are the thickness and relative permittivity of the dielectric substrate, respectively. Equation (4) implies that a thin dielectric substrate of high relative permittivity is desirable to obtain a maximum permittivity change according to the salinity in seawater. To this end, a silicon sheet is selected as a dielectric substrate which has 0.1 mm thickness and 11.9 relative permittivity. Using finite element method-based simulations to achieve maximum radiation efficiency, a chamber height ($d_{in}$) of 5 mm was obtained.

**Figure 3:**
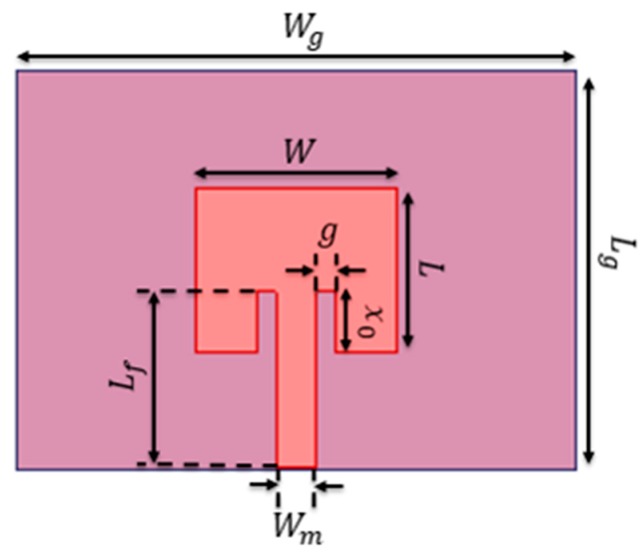
Top view of the feed inset patch antenna.

Since the resonance change according to salinities is relatively larger at higher frequency than the change at lower frequency, subtle changes in concentration can be measured with high accuracy and resolution at a higher resonance frequency. The proposed sensor has a linear resonance frequency dependency on salinity. However, the maximum resonance frequency should be placed at a detectable range. The proposed microstrip sensor has a higher resonance frequency at lower permittivity, and the relative permittivity of seawater decreases as temperature and salinity increases. Therefore, the sensor should be designed in such a way that the maximum resonance frequency ($f_{rmax}$) is placed at minimum permittivity ($\varepsilon_{rmin}$) obtained from the considered temperature and salinity range. By using $\varepsilon_{rmin}$ and $f_{rmax}$, width *(W*) and length (*L*) of the patch sensor can be obtained as follows [17]:

(5) $$W=\frac{c}{2f_{rmax}}\sqrt{\frac{2}{\varepsilon_{rmin}+1}}$$

(6) $$L=\frac{c}{2f_{rmax}\sqrt{\varepsilon_{reffm}}}-2\;\Delta L$$

(7) $$\Delta L=\frac{412d\left(\varepsilon_{reffm}+0.3\right)\left(\frac{W}{d}+0.264\right)}{\left(\varepsilon_{reffm}-0.258\right)\left(\frac{W}{d}+0.8\right)}$$

(8) $$\varepsilon_{reffm}=\frac{\varepsilon_{rmin}+1}{2}+\frac{\varepsilon_{rmin}-1}{2\sqrt{1+12\frac{d}{W}}}$$

where $\varepsilon_{reffm}$ represents the effective permittivity of $\varepsilon_{rmin}$, c is the speed of light, and ΔL is the extended length due to fringing effect. In the above equations, there is a linear resonance frequency dependency on the effective permittivity of layered structure, and the effective permittivity can be directly computed from Equation (4). For design simplicity, maximum frequency, range of temperature, and the salinity are selected as 3 GHz, 10–30 °C, and 20–35 ppt, respectively. The $\varepsilon_{rmin}$ is calculated for the maximum temperature and maximum salinity within the specified range, then $\varepsilon_{rmin}$ is 58.15 when temperature is 30 °C and salinity is 35 ppt according to Equations (2) and (4). The parameters of the patch calculated from Equations (5)–(8) are *W* of 9.2 mm and *L* of 4.5 mm. The patch parameters g, $x_{0}$, $W_{m}$, and $L_{f}$ are calculated by using [17]. The g and $x_{0}$ are parameters of the inset feed for efficient feeding. The parameters of the microstrip feed line $W_{m}$ and $L_{f}$ are required for impedance matching with 50 Ω microstrip line. By adjusting the sensor parameters including *W* and *L* with HFSS for full wave analysis, the final design parameters of the feed inset microstrip patch antenna are obtained and summarized as in Figure 3 and Table 1.

## 3. Sensor Fabrication and Experiment Setup

**Figure 4:**
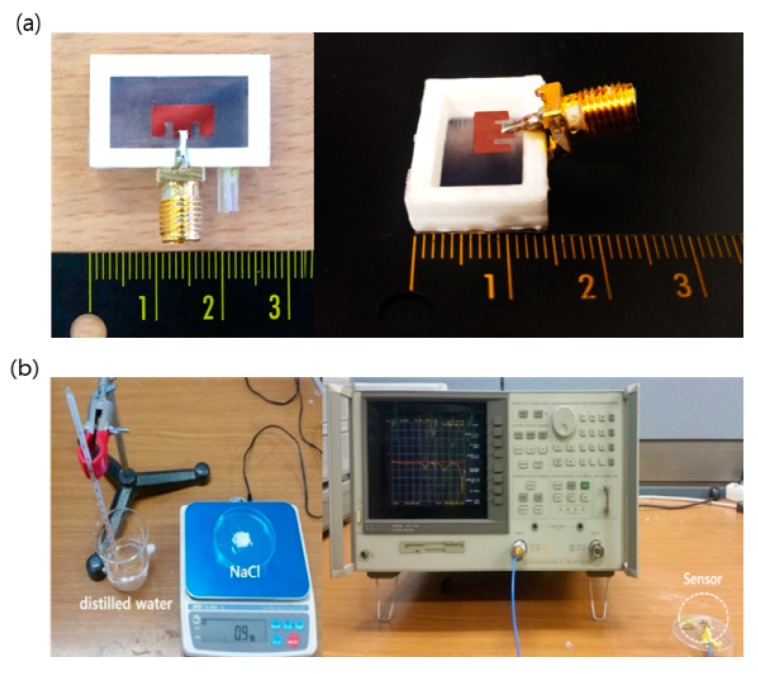
(**a**) Fabricated sensor; (**b**) Experiment setup.

The proposed sensor was fabricated by using a conductive copper coating material by MG Chemicals Inc. (Surry, BC, Canada), a 3D printer by 3Dison Inc. (Seoul, Korea), and a silicon substrate by K-silicon Inc. (Seoul, Korea). To make the patch and ground plane with conductive copper coating material, two masks were made by using a 3D printer. Then, the copper coating material was sprayed over the masks placed on silicon substrate with a coating having a thickness of a few hundred nanometers. After bonding both patches on the silicon substrates and attaching the patches on the chamber made by 3D printer, the sensor which has dimensions of 20 mm × 10 mm × 5 mm and can contain 1 mL of seawater was finally made. The manufactured sensor is shown in Figure 4a.

To measure the resonance frequency shift, the fabricated sensor was characterized by using a calibrated Agilent Technology vector network analyzer (VNA) (Agilent Technology, Santa Clara, CA, USA) connected with a 50 Ω transmission line and a 50 Ω RF SubMiniature version A (SMA) connector. (Agilent Technology, Santa Clara, CA, USA). To verify the resonance change to salinity determination, an experiment was performed by adding NaCl to distilled water. The experimental setup is shown in Figure 4b.

## 4. Results and Discussion

**Figure 5:**
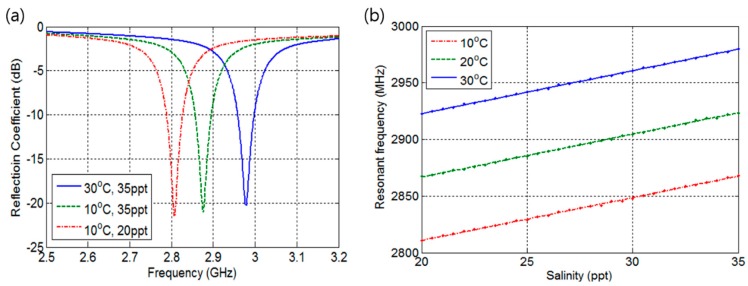
Simulation results. (**a**) Frequency response; (**b**) Resonant frequency according to salinity level.

**Figure 6:**
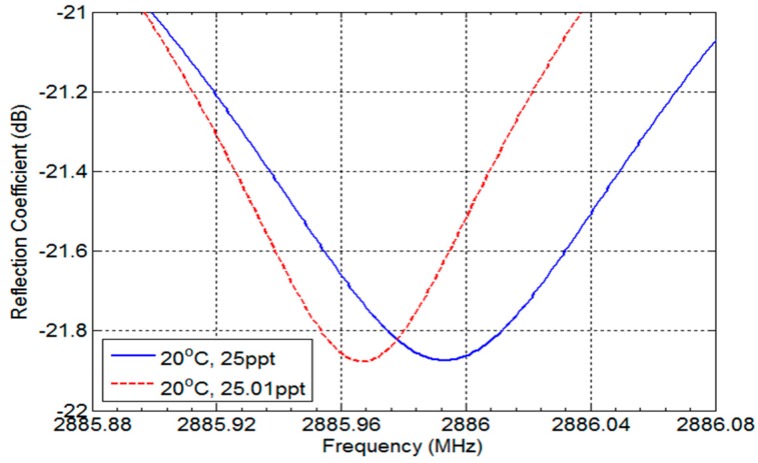
Simulation results for 0.01 ppt interval variation.

The ANSYS HFSS was utilized for full wave analysis of the proposed sensor design. By sweeping the frequency from 2.5 to 3.2 GHz, the reflection coefficients ($S_{11}$) according to salinity at fixed temperature were obtained as shown in Figure 5a. The Figure 5a shows the reflection coefficients at resonance frequencies obtained at a salinity of 20 ppt and 35 ppt at temperatures of 10 °C and 30 °C. The reflection coefficients were below −20 dB, which indicates that the load impedance of the design matches well with various seawater conditions. Figure 5b represents resonance frequencies to salinities at temperatures of 10 °C, 20 °C, and 30 °C, which are obtained with 0.5 ppt salinity increment. The blue, green, and red lines are approximated first-order interpolation functions with which the frequency separation—difference between resonance frequencies represented by blue and green or between green and red lines—of 57 MHz and maximum resonance frequency of 2980 MHz were obtained at a temperature of 30 °C. The estimated first-order interpolation function of resonance frequency ($f_{r}$) can be written as follows:

(9) $$f_{r}=3.79S+\left(2679+5.6T\right)$$

where S and T represent salinity and temperature, respectively. The interpolation error is 0.38 ppt, which is five times better than commercial salinity determination sensors [5,6]. Furthermore, the proposed sensor can detect a 0.01 ppt change in salinity by differentiating resonance frequency change with 37.9 kHz resolution. The resonance frequencies at a temperature of 20 °C are shown in Figure 6, which exhibits twice better resolution than sensors in [5,6].

**Figure 7:**
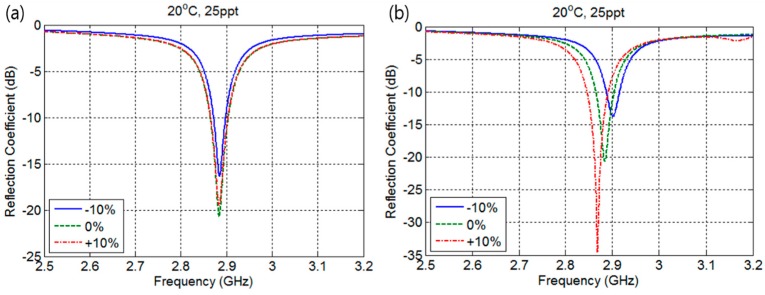
Frequency response according to fabrication error: (**a**) Fabrication error for patch width *W*; (**b**) Fabrication error for patch length *L*.

**Figure 8:**
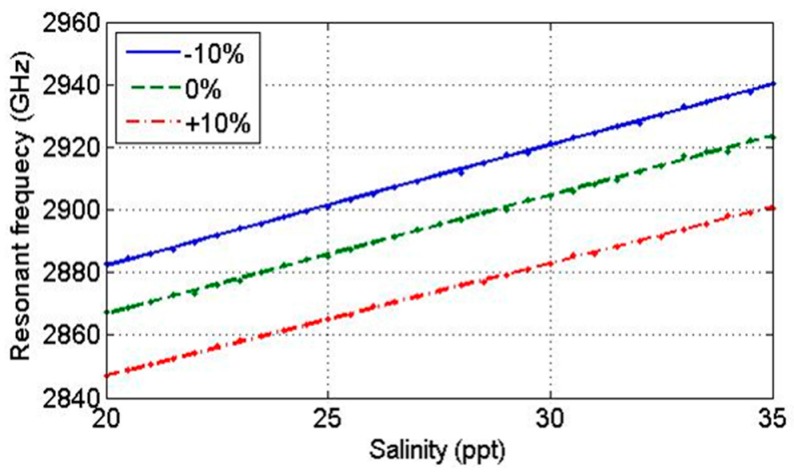
Resonant frequency according to salinity level by fabrication error.

Next, the proposed sensor performance according to fabrication error was analyzed. Fabrication error of ±10% is considered in both parameters *W* and *L*, which results in W=9.2±0.92 mm and L=4.5±0.45 mm. The reflection coefficient changes according to errors in patch W and L at temperature of 20 °C and salinity of 25 ppt are shown in Figure 7a,b, respectively. The fringing field is formed at the edges of the patch and ground plane in the direction of the microstrip line; therefore, it can be expected that the error in *W* does not affect resonance frequency, but on radiation efficiency. However, fabrication error in *L* affects resonance frequency, which shifts to lower frequency with larger values of *L* and to higher frequency with smaller *L*. The reflection coefficient becomes 15 dB smaller as error in *L* increases. Figure 8 represents first-order interpolation functions of resonance frequencies changes to ±10% error in *L* with 0.5 ppt salinity increment. It can be observed that the frequency separation between 20 and 35 ppt salinities remains the same as 57 MHz, whereas ±16 MHz frequency bias occurs.

The performance of the proposed sensor is compared with existing sensors and summarized as in Table 2. The accuracy and resolution of the proposed sensor are better than the portable sensor, but poorer than CTD. However, the proposed sensor can determine salinity at the sea surface, which is not possible with CTD, and it can be fabricated with low cost and in compact size.

**Figure 9:**
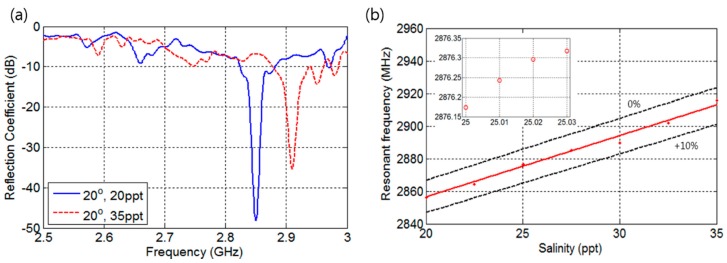
Experimental results: (**a**) Reflection coefficients of the proposed sensor; (**b**) Resonant frequency according to salinity.

The measured reflection coefficients of the fabricated sensor at temperature of 20 °C in the frequency range of 2.5 to 3 GHz are shown in Figure 9. As shown in Figure 9a, the measured reflection coefficients for different salinities are smaller than or equal to −35 dB. The measured reflection coefficients to salinity with 2.5 ppt increment are shown in Figure 9b, which reveals that the fabricated sensor has approximately 5% fabrication error in *L*. This small fabrication error results in a 59 MHz frequency separation, which is 2 MHz greater than the simulation. The resonance frequency change to 0.01 ppt is shown in the inset of Figure 9b and shows an average frequency separation of 48 kHz, which is 26.6% better separation than simulation results of 37.9 kHz.

## 5. Conclusions

A compact microstrip patch sensor is proposed to measure salinity in seawater. The sensor is designed to have resonant frequency less than 3 GHz between 20 and 30 ppt salinity. The performance of the sensor design was verified through HFSS simulation. The simulation results showed 37.9 kHz resonance frequency separation with 0.01 ppt salinity resolution and maximum −20 dB reflection coefficients at the resonant frequency. The proposed sensor was fabricated by using conductive copper spray coatings, silicon substrate, and 3D printer. The patch and ground plane attached to the silicon substrate were bonded to a 5 mm-high chamber made by 3D printer that contained 1 mL seawater. To verify the proposed sensor performance, experiments were conducted by adding different amounts of NaCl to distilled water. Experimental results showed that the fabricated sensor had an average of 48 kHz frequency separation for 0.01 ppt salinity resolution and maximum −35 dB reflection coefficient. These results prove that we can easily measure salinity in seawater with the proposed low-cost senor. This work could be extended to a self-contained system for measuring salinity in marine life.

## Figures and Tables

**Table 1:** Dimension of the feed inset microstrip patch antenna.

Parameters	Unit (mm)	Parameters	Unit (mm)
$W_{g}$	20	$W_{m}$	3
$L_{g}$	10	$L_{f}$	2.2
W	8.7	g	0.87
L	4.1	$x_{0}$	1.5

**Table 2:** Performance and characteristics of salinity sensors. CTD: conductivity-temperature-depth sensor.

	Microstrip Sensor	Portable Sensor [6]	CTD [21]
Accuracy (uncertainty)	0.38 ppt	2 ppt	0.05 ppt
Resolution	0.01 ppt	0.02 ppt	0.004 ppt
Price	Low	Low	High
Specimen volume	Small	Small	Large
Surface measurement	Yes	Yes	No

## References

[1] Bennett A.S. (1976). Conversion of in situ measurements of conductivity to salinity. Deep Sea Res. Oceanogr. Abstr..

[2] Hooker S.K., Boyd I.L. (2003). Salinity sensors on seals: Use of marine predators to carry CTD data loggers. Deep Sea Res. Part I.

[3] Knowles C.E. (1974). Salinity determination from use of CTD sensors. J. Phys. Oceanogr..

[4] Reul N., Fournier S., Boutin J., Hernandez O., Maes C., Chapron B., Alory G., Quilfen Y., Tenerelli J., Morisset S. (2014). Sea surface salinity observations from space with the SMOS satellite: A new means to monitor the marine branch of the water cycle. Surv. Geophys..

[5] Vernier Software & Technology, Sal-bta. https://www.vernier.com.

[6] Center for Microcomputer Application, Salinity Sensor Bt78i. http://www.cma-science.nl.

[7] Parra L., Sendra S., Lloret J., Bosch I. (2015). Development of a conductivity sensor for monitoring groundwater resources to optimize water management in smart city environments. Sensors.

[8] Parra L., Ortuño V., Sendra S., Lloret J. Two new sensors based on the changes of the electromagnetic field to measure the water conductivity. Proceedings of the Seventh International Conference on Sensor Technologies and Applications (SENSORCOMM 2013).

[9] Cook B.S., Cooper J.R., Tentzeris M.M. (2013). An inkjet-printed microfluidic RFID-enabled platform for wireless lab-on-chip applications. IEEE Trans. Microw. Theory Tech..

[10] Jaworek A., Krupa A. (2004). Gas/liquid ratio measurements by RF resonance capacitance sensor. Sens. Actuators A Phys..

[11] Cheng E.M., Fareq M., Shahriman A.B., Mohd Afendi R., Lee Y.S., Khor S.F., Tan W.H., Nashrul Fazli M.N., Abdullah A.Z., Jusoh M.A. (2014). Development of microstrip patch antenna sensing system for salinity and sugar detection in water. Int. J. Mech. Mechatron. Eng. IJMME-IJENS.

[12] Ramhat-Samii Y., Kona K., Manteghi M. Microstrip Antenna for Remote Sensing of Soil Moisture and Sea Surface Salinity. https://www.techbriefs.com/component/content/article/1059-gdm/tech-briefs/5378-npo-44470-96520914?Itemid=690.

[13] Lide D.R., Haynes W.M. (2009). CRC Handbook of Chemistry and Physics.

[14] Wentz F.J., Meissner T. (1998). AMSR OCEAN Algorithm, Algorithm Theoretical Basis Document. Remote Sens. Syst. Tech. Rep..

[15] Meissner T., Wentz F.J. (2004). The complex dielectric constant of pure and sea water from microwave satellite observations. IEEE Trans. Geosci. Remote Sens..

[16] Stogryn A.P., Bull H.T., Rubayi K., Iravanchy S. (1995). The Microwave Permittivity of Sea and Fresh Water.

[17] Constantine A.B. (2016). Antenna theory: Analysis and design. Microstrip Antennas.

[18] Pozar D.M. (1992). Microstrip antennas. Proc. IEEE.

[19] Nagendra P., Aparna G., Soni C. (2015). Analysis of multilayer stacked microstrip patch antenna for bandwidth enhancement. Int. J. Innov. Res. Sci. Eng. Technol..

[20] Petosa A., Simons N., Siushansian R., Ittipiboon A., Cuhaci M. (2000). Design and analysis of multisegment dielectric resonator antennas. IEEE Trans. Antennas Propag..

[21] Sea-Bird Electronics, Sbe 911 Plus CTD. http://www.seabird.com.

